# Study of the oxygen vacancy influence on magnetic properties of Fe- and Co-doped SnO_2_ diluted alloys

**DOI:** 10.1186/1556-276X-7-540

**Published:** 2012-09-28

**Authors:** Pablo D Borges, Luisa M R Scolfaro, Horacio W Leite Alves, Eronides F da Silva, Lucy V C Assali

**Affiliations:** 1 Instituto de Ciências Exatas e Tecnológicas, Universidade Federal de Viçosa - CRP, Rio Paranaíba, Minas Gerais, CP 38810-000, Brazil; 2 Department of Physics, Texas State University, San Marcos, TX, 78666, USA; 3 Departamento de Ciências Naturais, Universidade Federal de São João Del Rei, CP 110, São João Del Rei, Minas Gerais, 36301-160, Brazil; 4 Departamento de Fisica, Universidade Federal de Pernambuco, Recife, Pernambuco, 50670-901, Brazil; 5 Instituto de Fisica, Universidade de São Paulo, São Paulo, 05315-970, Brazil

**Keywords:** Tin dioxide, Diluted magnetic semiconductors, Magnetic properties, *ab initio* calculations, Electronic structure

## Abstract

Transition-metal (TM)-doped diluted magnetic oxides (DMOs) have attracted attention from both experimental and theoretical points of view due to their potential use in spintronics towards new nanostructured devices and new technologies. In the present work, we study the magnetic properties of Sn_0.96_TM_0.04_O_2_ and Sn_0.96_TM_0.04_O_1.98_(*V*_O_)_0.02_, where TM = Fe and Co, focusing in particular in the role played by the presence of O vacancies nearby the TM. The calculated total energy as a function of the total magnetic moment per cell shows a magnetic metastability, corresponding to a ground state, respectively, with 2 and 1 μ_B_/cell, for Fe and Co. Two metastable states, with 0 and 4 μ_B_/cell were found for Fe, and a single value, 3 μ_B_/cell, for Co. The spin-crossover energies (*E*_S_) were calculated. The values are *E*_S_^0/2^ = 107 meV and *E*_S_^4/2^ = 25 meV for Fe. For Co, *E*_S_^3/1^ = 36 meV. By creating O vacancies close to the TM site, we show that the metastablity and *E*_S_ change. For iron, a new state appears, and the state with zero magnetic moment disappears. The ground state is 4 μ_B_/cell instead of 2 μ_B_/cell, and the energy *E*_S_^2/4^ is 30 meV. For cobalt, the ground state is then found with 3 μ_B_/cell and the metastable state with 1 μ_B_/cell. The spin-crossover energy *E*_S_^1/3^ is 21 meV. Our results suggest that these materials may be used in devices for spintronic applications that require different magnetization states.

## Background

Nowadays, dilute magnetic oxides (DMOs) are potential candidates for both spintronic devices and nanodevices applications. Although the existence of room temperature ferromagnetism (FM) in transition metal (TM)-doped SnO_2_ has been reported, the origin of the FM is still controversial. There are indications that the FM comes from different sources, metallic clusters, secondary phases, or is due to a free carrier-mediated mechanism in the bulk. The presence of oxygen vacancies is systematically related to the observed ferromagnetic state. These systems are good candidates to obtain materials with a half-metallic behavior, with 100% spin-polarized carriers at the Fermi level.

Tin dioxide (SnO_2_) doped with transition metals (TMs) have been extensively investigated recently due to the resulting important magnetic properties [1-6]. The ferromagnetic behavior has been observed at room temperature in Cr-, Mn-, Fe- and Co-doped SnO_2_ DMO systems [7-12], indicating the potential of such systems for spintronic applications. It has also been observed that the presence of oxygen vacancies appears to be required for producing FM in DMOs, such as, e.g., in Co-doped ZnO [13], in Co-doped TiO_2_[14,15], in Fe- and Co-doping in In_2_O_3_[16,17], and in Fe-, Co-, and Cr- doped SnO_2_[7,18,19]. A theoretical model proposed by Coey et al. to interpret the FM in these semiconducting oxides requires the existence of oxygen vacancies in close proximity to TM sites in order to maintain the charge neutrality [20]. For Cr-doped SnO_2_ nanoparticles, the obtained FM behavior is limited by a maximum doping concentration *x*_L_ which has a strong relation with structural changes revealed from X-ray diffraction measurements [7]. The presence of oxygen vacancies in these Sn_1 − *x*_TM_*x*_O_2_ samples, in which the TM concentrations *x* varies from 0% to 10%, has been detected by electron paramagnetic resonance experiments [19]. Many efforts have been made in attempt to characterize and understand the mechanisms involved in the ferromagnetic behavior observed in such systems.

In this work, we study the oxygen vacancy influence on magnetic properties of the Fe- and Co-doped SnO_2_ diluted alloys. First, the systems Sn_1 − *x*_MT_*x*_O_2_, for *x* = 0.04, were studied through *ab initio* electronic structure calculations performed within the spin density functional theory. The concentration of *x* = 0.04 corresponds to a typical experimental value. Second, an oxygen vacancy nearest neighbor to the TM atom in the alloys was introduced. Sn_1 − *x*_TM_*x*_O_2 − *y*_(*V*_O_)_*y*_ systems, with *x* = 0.04 and *y* = 0.02, and its consequences for the magnetic behavior of these systems were considered. Finally, an investigation about the magnetic metastability was done, and the spin-crossover phenomenon was studied. A metamagnetic state is the key underlying conceptual mechanism for storage, memory, and display device and nanodevice applications [21], and this work shows that DMO materials based on Fe- and Co-doped SnO_2_ taking into account the oxygen vacancy influence could be engineered to display different stable magnetized states.

## Methods

The calculations were based on the spin density functional theory. We employed the projector augmented wave method implemented in the Vienna ab-initio simulation package (VASP-PAW) [22,23]. The exchange-correlation potential used was the generalized gradient approximation in the Perdew, Burke, and Ernzerhof [24] approach. The method has been previously used to study the structural and electronic properties of bulk rutile SnO_2_[25]. The valence electronic distributions for the PAWs representing the atoms were Sn 4*d*^10^5*s*^2^5*p*^2^, Fe 3*d*^7^4*s*^1^, Co 3*d*^7^4*s*^2^, and O 2*s*^2^2*p*^6^. The onsite correction Hubbard *U* for Co- and Fe *d* orbitals was not considered. Previous calculations for Cr doping SnO_2_ taking into account a *U* correction show no major changes in our conclusions. Scalar relativistic effects were taken into account. To describe the alloys, we used a 72-atom supercell (24 Sn and 48 O atoms) and a 4 × 4 × 4 mesh of Monkhorst-Pack k-points for integration in the Brillouin zone. All the calculations were done with a 490-eV energy cutoff in the plane-wave expansions, and the systems were fully relaxed until the residual forces on the ions were less than 10 meV/Å.

## Results and discussion

We studied the systems Sn_0.96_TM_0.04_O_2_ and the Sn_0.96_TM_0.04_O_1.98_(*V*_O_)_0.02_ (with TM = Fe and Co) with the oxygen vacancy as the TM nearest neighbor. In both cases, a single tin atom was substituted with a TM atom in the 72-atom supercell, simulating the *x* = 0.04 impurity and *y* = 0.02 oxygen vacancy concentrations. For all cases, the total energy was calculated for several magnetic moment values.

It was recently shown by us [26] that when a Sn atom is replaced by a chromium atom in SnO_2_, a high-spin (HS) ground state with a magnetic moment *m* = 2 μ_B_/cell and a low-spin state (LS) with a magnetic moment *m* = 0 μ_B_/cell are obtained. For this case, a spin crossover becomes possible with an energy barrier of 114 meV calculated for the transition from *m* = 0 to 2 μ_B_/cell. When an oxygen vacancy (out of the six first neighbors of Cr) is considered, the behavior of the total energy versus the magnetic moment per cell showed the appearance of a second HS configuration, with magnetic moment *m* = 4 μ_B_/cell and an energy barrier of 32 meV relative to the 2 μ_B_/cell state. The ground state, however, remains as the 2 μ_B_/cell magnetic moment HS state. The energy barrier for the *m* = 0 to 2 μ_B_/cell transition was reduced to 27 meV. Comparing this value with the one for this barrier in the DMO without the vacancy (114 meV), a drastic transition for the DMO without the vacancy of 114 meV, a drastic reduction by about 75% is observed.

**Figure 1:**
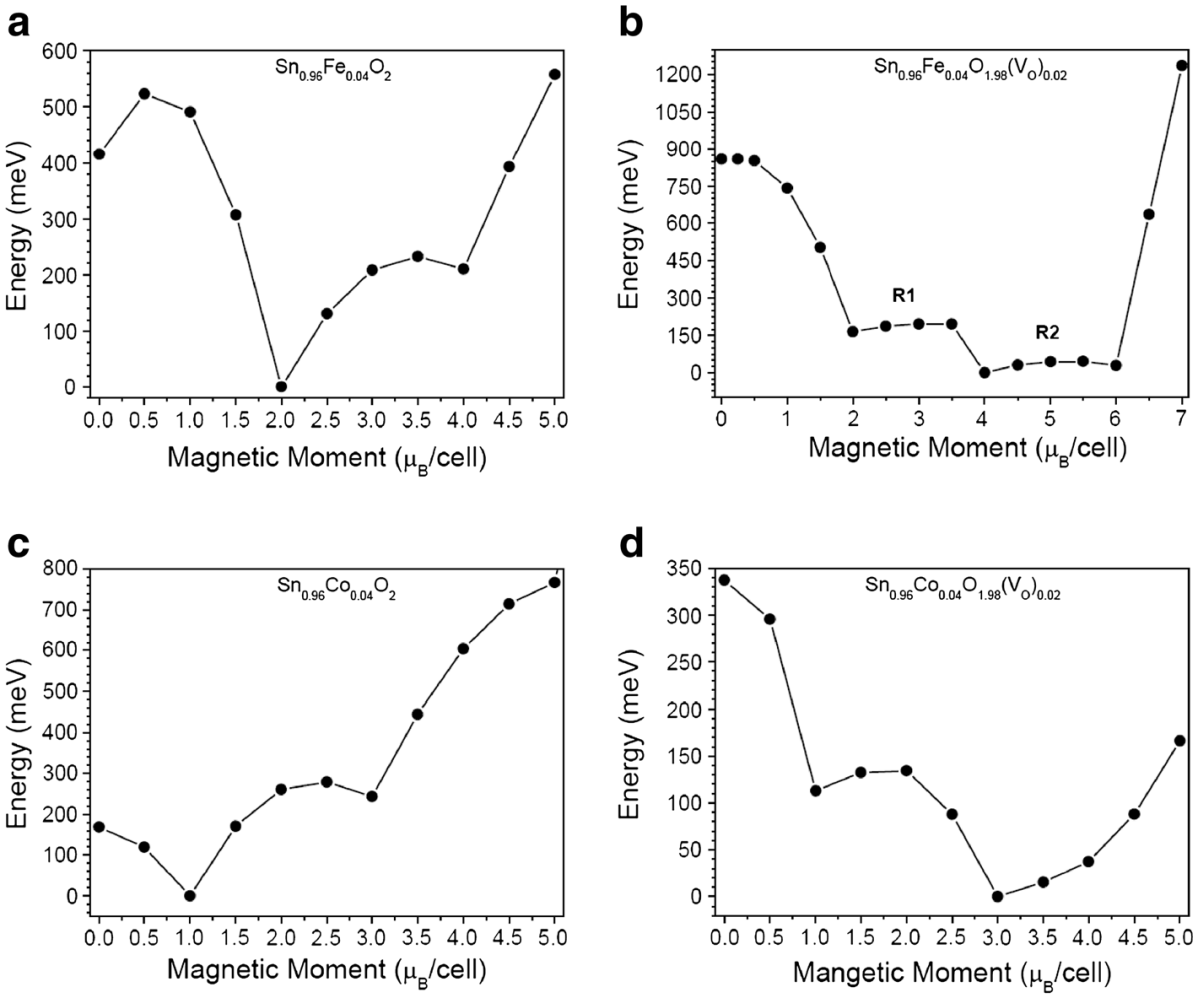
** Total energy vs. magnetic moment per cell.** For (**a**) Sn_0.96_Fe_0.04_O_2_, (**b**) Sn_0.96_Fe_0.04_O_1.98_(*V*_O_)_0.02_, (**c**) Sn_0.96_Co_0.04_O_2_, and (**d**) Sn_0.96_Co_0.04_O_1.98_(*V*_O_)_0.02_. The total energy of the high-spin ground state is set to zero.

We show here that if a single Sn atom is substituted by a Fe or a Co impurity, the magnetic metastability observed for Cr-doped SnO$_2$ diluted alloys also occurs. Figure 1a,b shows the total energy behavior for the Sn_0.96_Fe_0.04_O_2_ and Sn_0.96_Fe_0.04_O_1.98_(*V*_O_)_0.02_ diluted magnetic alloys, respectively, in function of magnetic moment/cell. A ground state with a magnetic moment *m* = 2 μ_B_/cell and two metastable states with magnetic moments of *m* = 0 and *m* = 4 μ_B_/cell were observed for the system without the oxygen vacancy. The crossover energy barrier for the transition from *m* = 0 to 2 μ_B_/cell is *E*_S_^0/2^ = 107 meV, and for the transition from *m* = 4 to 2 μ_B_/cell is *E*_S_^4/2^ = 25 meV. When the oxygen vacancy is considered, *m* = 0 μ_B_/cell is no longer a metastable state; however, new states appear with magnetic moment varying in the range 2 ≤ *m* ≤ 6. The ground state occurs at *m* = 4 μ_B_/cell, and two almost flat regions between 2 ≤ *m* ≤ 3.5 μ_B_/cell (region R1) and 4 ≤ *m* ≤ 6 μ_B_/cell (region R2) are seen. Energy barriers of *E*_S_^2/4^ = 30 meV for the transition from *m* = 2 to 4 μ_B_/cell and *E*_S_^6/4^ = 16 meV for the transition from *m* = 4 to 6 μ_B_/cell were observed. Figure 1c,d shows the total energy versus magnetic moment for Sn_0.96_Co_0.04_O_2_ and Sn_0.96_Co_0.04_O_1.98_(*V*_O_)_0.02_ alloys, respectively. The ground state for the system without the oxygen vacancy is at *m* = 1 μ_B_/cell, and a magnetic metastable at *m* = 3 μ_B_/cell is seen. The energy barrier of *E*_S_^3/1^ = 36 meV for the transition from *m* = 3 to 1 μ_B_/cell was obtained. For the Co-doped SnO_2_ with an oxygen vacancy, we obtain a change in the magnetic metastable states: the ground state is at *m* = 3 μ_B_/cell and a magnetic metastable at *m* = 1 μ_B_/cell is seen, with a crossover barrier energy of *E*_S_^1/3^ = 21 meV from *m* = 1 to 3 μ_B_/cell.

A better understanding of the behavior for the magnetic moment can be obtained if we analyze the oxidation states of the TM and of the neighbor atoms around it. For the Sn_0.96_Fe_0.04_O_2_ alloy, the Fe^4+^ (3*d*^4^) impurity replacing Sn^4+^ allows the magnetic states *m* = 0, 2, and 4 μ_B_/cell. In this case, the neighborhood does not contribute to the magnetization of the system. When an oxygen vacancy is taken into account, the impurity state changes from Fe^4+^ to Fe^3+^ (3*d*^5^) and to Fe^2+^ (3*d*^6^), and the neighboring atoms contribute to the magnetic moment. For *m* = 2 μ_B_/cell, the oxidation state is Fe^3+^ where four spin-up and one spin-down electrons from Fe plus one spin-down electron arising from the neighboring atoms allow for the metastable state (with *m* = 2 μ_B_/cell). For the *m* = 4 and 6 μ_B_/cell, the oxidation state is Fe^2+^ where five spin-up and one spin-down electrons from Fe originate the state *m* = 4 μ_B_/cell, while five spin-up and one spin-down electrons from Fe plus two spin-up electrons arising from the neighboring atoms allow the metastable state with *m* = 6 μ_B_/cell. These findings are in agreement with the experimental data [27-30].

Likewise, for the Sn_0.96_Co_0.04_O_2_ dilute magnetic alloy, the Co^4+^ (3*d*^5^) and Co^3+^ (3*d*^6^) impurity replacing Sn^4+^ give rise to the magnetic states *m* = 1 and 3 μ_B_/cell, respectively. For *m* = 1 μ_B_/cell, three spin-up and two spin-down electrons from cobalt and for *m* = 3 μ_B_/cell four spin-up and two spin-down electrons from cobalt plus one spin-up electron arising from the neighboring atoms allow this metastable state. The states Co^3+^ (3*d*^6^) and Co^2+^ (3*d*^7^) are possible when an oxygen vacancy is taken into account for *m* = 1 and 3 μ_B_/cell, respectively. For *m* = 1 μ_B_/cell, four spin-up and two spin-down electrons plus one spin-down electron from neighboring atoms are involved. For *m* = 3 μ_B_/cell, five spin-up and two spin-down electrons from cobalt give rise to this metastable state. Experimental studies have confirmed the incorporation of Co^2+^ cations into the rutile SnO_2_ lattice [31].

**Figure 2:**
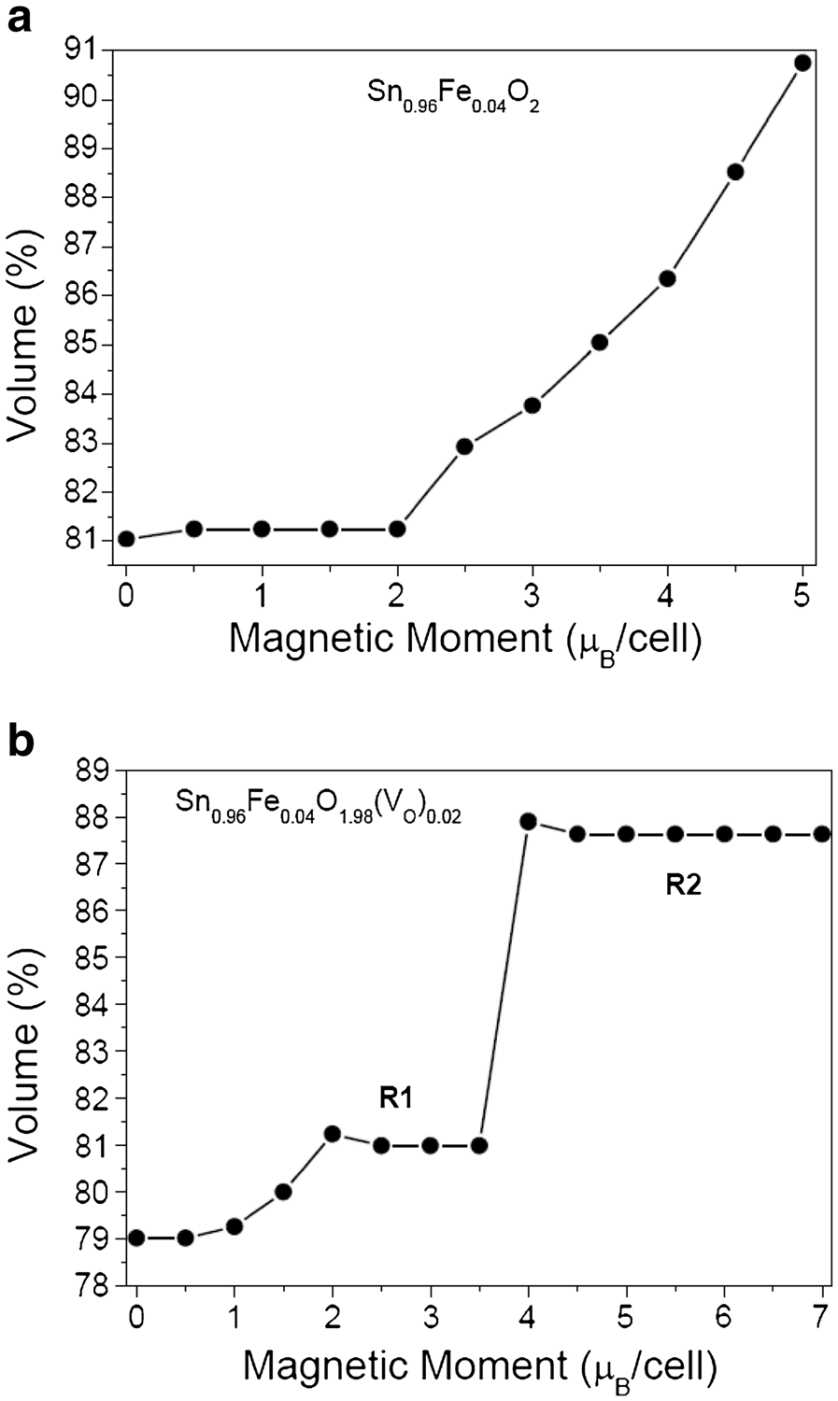
** Relative volume (%) around the iron atom vs. magnetic moment per cell.** For (**a**) Sn_0.96_Fe_0.04_O_2_ and (**b**) Sn_0.96_Fe_0.04_O_1.98_(*V*_O_)_0.02_.

As observed previously for the chromium impurity [26], for iron and cobalt we also observe a relationship between the structural modification around the TM atom, due to the electronic and ionic relaxations, and the occurrence of the magnetic metastability. If we consider a spherical volume involving the TM whose radius is an average distance between the TM and the six oxygen nearest neighbors, our calculations showed that, after full relaxation, the corresponding volume is reduced. For Co, the volume is reduced by about 16% for the HS state (2 μ_B_/cell) and by 17.5% for the LS state (0 μ_B_/cell). Considering the oxygen vacancy, for the HS states (2 and 4 μ_B_/cell) the volume reductions, after full relaxed calculations, were 20% and 22%, respectively, while for the LS state (0 μ_B_/cell), it was 31%. For LS configurations, the presence of an oxygen vacancy allows greater relaxations which reduce the total energy of the system lowering the energy barrier for the crossover. As shown in Figure 2a for Fe, the volume is reduced by about 19% for the states *m* = 0 and 2 μ_B_/cell, and by 14% for the state *m* = 4 μ_B_/cell. If the oxygen vacancy is present, the volume around the Fe impurity is reduced by about 19% for the magnetic moment between 2 and 3.5 μ_B_/cell, region R1, and by 12% for the range between 4 and 6 μ_B_/cell, region R2, shown in Figure 2b, both corresponding to an almost flat region. For the cobalt impurity, the volume around Co is reduced by about 19% for the state *m* = 1 μ_B_/cell and by 15% for the state *m* = 3 μ_B_/cell. Considering an oxygen vacancy near the Co atom, the volume is reduced by about 19% for the state *m* = 1 μ_B_/cell and by 11% for the state *m* = 3 μ_B_/cell. The observed magnetic metastability in the DMOs studied here is attributed to a structural modification (relaxations) around the TM impurity. The presence or absence of magnetism in this case is determined by a competition between intra-atomic exchange interactions and inter-atomic electronic motion due to the crystalline field [32]. Therefore, the occurrence of the LS and HS states depends on the effective balance between these two interaction fields. The LS state occurs when the crystal field splitting is larger than the intra-atomic exchange splitting (lowest volume); otherwise, the HS state is the ground state (highest volume). 

**Figure 3:**
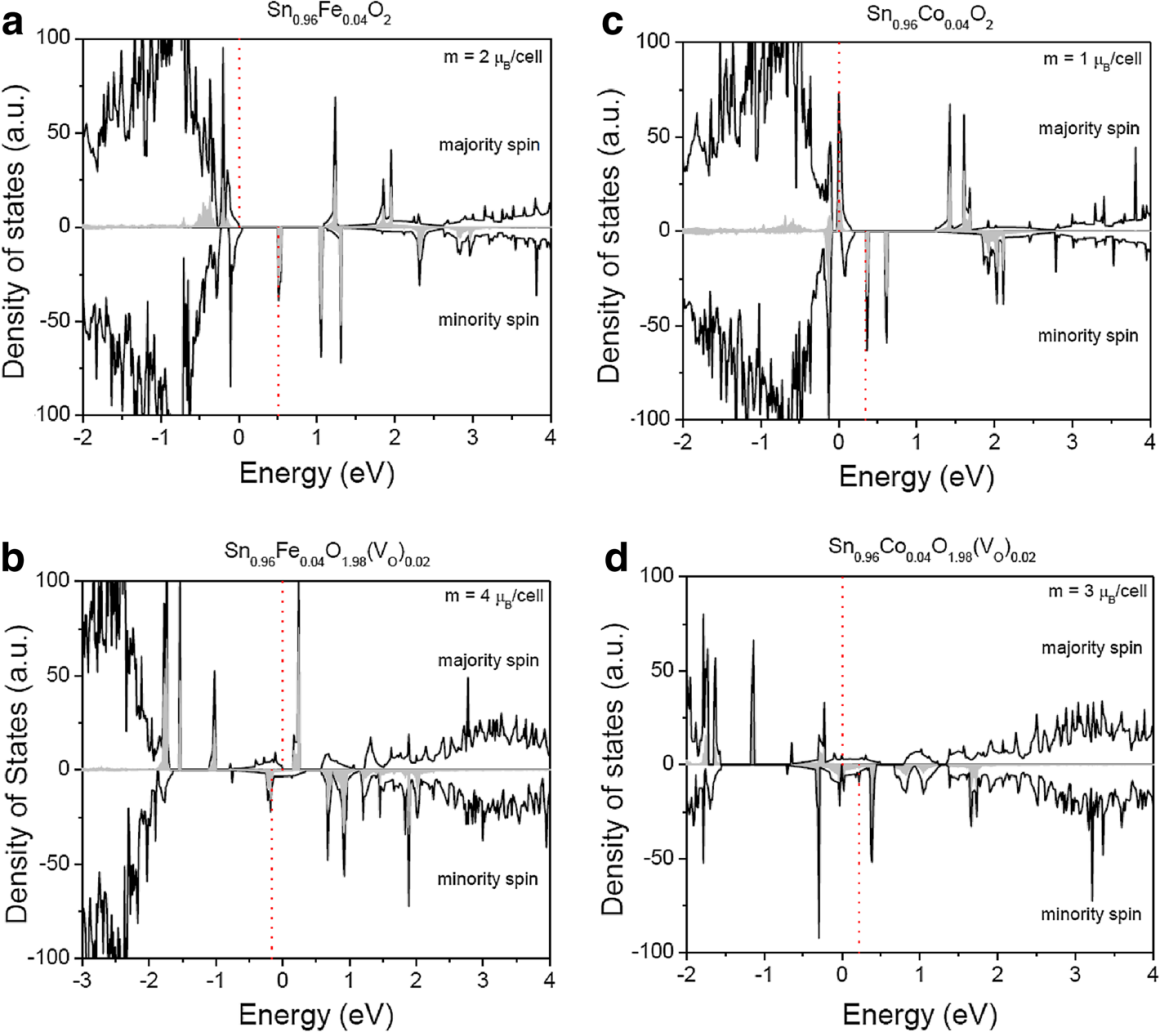
** The ground state total (black lines) and projected (gray shaded areas) density of states.** The transition metal 3*d*-derived states for the majority and minority spins for the ground state magnetic moment are shown for (**a**) Sn_0.96_Fe_0.04_O_2_, (**b**) Sn_0.96_Fe_0.04_O_1.98_(*V*_O_)_0.02_, (**c**) Sn_0.96_Co_0.04_O_2_, and (**d**) Sn_0.96_Co_0.04_O_1.98_(*V*_O_)_0.02_. The vertical dashed lines correspond to the highest occupied energy level for the majority and minority spins.

The ground state total and projected density of states of the Fe 3*d* orbital are shown in Figure 3a,b for Sn_0.96_Fe_0.04_O_2_ and Sn_0.96_Fe_0.04_O_1.98_(*V*_O_)_0.02_ alloys, respectively. The vertical lines represent the energy values of the highest occupied states for the majority and minority spins. For both cases, the iron 3*d*-derived states are found to lie in the gap region, showing a half-metallic behavior. For Sn_0.96_Co_0.04_O_2_ and Sn_0.96_Co_0.04_O_1.98_(*V*_O_)_0.02_ alloys, the DOS are shown in Figure 3c,d, respectively. For all cases, we observe a strong character arising from the 3*d* orbital near the gap region. This feature is also responsible for the half-metallic behavior displayed by the DMOs studied here.

## Conclusions

The influence of the oxygen vacancy in the magnetic and electronic properties of iron and cobalt as impurities in a DMO configuration in rutile SnO_2_ was studied using first principle calculations performed within the spin-density functional theory. A magnetic metastability was observed for both impurity cases. Energy barriers were obtained for the spin crossover between the *m* = 0 and 2 μ_B_/cell and between the *m* = 2 and 4 μ_B_/cell states in Sn_0.96_Fe_0.04_O_2_. For Sn_0.96_Co_0.04_O_2_, the observed magnetic metastability and energy barrier were obtained for the spin crossover between the *m* = 3 and 1 μ_B_/cell states.

When an oxygen vacancy is considered as one of the six first neighbors to the Fe and Co impurities in these alloys (Sn_0.96_Fe_0.04_O_1.98_(V_O_)_0.02_ and Sn_0.96_Co_0.04_O_1.98_(V_O_)_0.02_), a considerable modification is observed in the magnetic metastability behavior, with new allowed states appearing for iron. For the cobalt impurity, the presence of an oxygen vacancy promoted a ground state exchange between the magnetic state 1 and 3 μ_B_/cell. This behavior is attributed to the relative contributions of the intra-atomic exchange interaction effects and the inter-atomic electron motion effects due to the crystalline field, which are responsible for the relaxations around the TM impurities. Finally, to manipulate the electron spin opens new possibilities to engineering new spintronic devices, and TM-doped DMOs are potential candidates to represent a new kind of material, which display magnetic metastability, SCO phenomena, and a half-metallic behavior.

## Competing interests

The authors declare that they have no competing interests.

## Authors’ contributions

PDB performed the *ab initio* calculations, participated in the analysis, and drafted the manuscript. LMRS and PDB conceived of the study. HWLA, EFS, LVCA, and LMRS participated in the analysis and in the production of the final version of the manuscript. All authors read and approved the final manuscript.

## Authors’ information

PDB is an assistant professor at the Universidade Federal de Viçosa. LMRS is a senior lecturer and research professor at Texas State University-San Marcos. HWLA is an associate professor at Universidade Federal de São João del Rei. EFS is an associate professor at Universidade Federal de Pernambuco. LVCA is an associate professor at Universidade de São Paulo.
